# Determinants of Rule‐Breaking in Adolescence

**DOI:** 10.1002/jad.70031

**Published:** 2025-08-12

**Authors:** Karlijn Hoyer, Jelle Sijtsema, Christoph Kogler, Wouter van den Bos, Lucas Molleman

**Affiliations:** ^1^ Psychology University of Amsterdam Amsterdam the Netherlands; ^2^ Educational Science University of Groningen Groningen the Netherlands; ^3^ Developmental Psychology Tilburg University Tilburg the Netherlands; ^4^ Social Psychology Tilburg University Tilburg the Netherlands

## Abstract

**Introduction:**

Learning to navigate the many rules that regulate behavior in the adult world is a key developmental goal in adolescence. Yet, adolescents are notorious rule‐breakers. In this paper, we use behavioral experiments to examine how adolescent rule‐breaking is impacted by situational ambiguity and social influence.

**Methods:**

Younger (11−15 year‐olds, *N* = 296) and older adolescents (16−19 year‐olds, *N* = 333) recruited from high schools in the Netherlands (44% male, 54% female, 2% other) completed incentivized tasks. Each task involved a red traffic light, and participants were told that the rule was to wait until it turned green. However, by breaking the rule and crossing before the green light, they could increase their rewards. The tasks varied in two factors: situational ambiguity (a yellow light appearing before green) and peer behavior (observing peers either following or breaking the rule).

**Results:**

Adolescents' rule‐breaking is highly sensitive to ambiguity and peer influence. Rule‐breaking strongly increases when there is wiggle room to interpret a situation in self‐serving ways, and when observing rule‐breaking by peers. The magnitude of these effects does not differ between younger and older adolescents, but overall, older adolescents tend to break the rule more often than younger adolescents. Moreover, older adolescents' injunctive norms change more in response to others' behavior. Exploratory analyses indicate that boys break the rule more often and that girls are substantially more sensitive to peer influence than boys.

**Conclusions:**

Our experimental results indicate that interventions aimed at curbing adolescents' rule‐breaking may benefit from reducing situational ambiguity and promoting exposure to positive peer behavior.

## 1 Introduction

Adolescents navigate a web of rules in their daily lives, ranging from school dress codes to parental curfews and societal expectations like gender roles. These rules are crucial for their development, providing structure and guiding socialization (e.g., Steinberg and Morris 2001). Yet, adolescents are notorious rule‐breakers (Eaton et al. 2008), in some cases even more so than adults (Icenogle and Cauffman 2021; Shulman et al. 2013). They frequently engage in underage drinking, drug use, and unprotected sex (Coleman and Cater 2005). This behavior may often serve immediate self‐interest, but can have legal consequences, carry health risks, and lead to negative outcomes later in life (Dahl et al. 2018; Windle and Zucker 2010). It is therefore important to know when and why adolescents break rules (Rosenstein 2009). Understanding underlying motivations, the influence of (social) context, and developmental trajectories of these behaviors can inform prevention and intervention efforts aimed at promoting positive youth development, reducing risk factors for delinquency, and fostering healthy decision‐making skills among adolescents.

Although rule‐breaking is central to adolescence, most research in this area has focused on risk‐taking. While these behaviors can overlap, they are distinct: rule‐breaking involves defying laws or social norms, while risk‐taking often involves seeking potentially dangerous experiences without necessarily violating rules (e.g., extreme sports like rock climbing or skydiving). At the same time, many rule‐breaking behaviors inherently involve elements of risk‐taking, such as stealing or tagging public spaces, which blur the line between the two categories. The risk‐taking literature highlights uncertainty and peer influence as key drivers of adolescent behavior (Caskey and Anfara 2014; Ciranka and van den Bos 2021; Reiter et al. 2021; Steinberg and Monahan 2007). Adolescents face novel environments that heighten uncertainty, and navigating these involves testing boundaries and taking risks (Romer and Hennessy 2007). Contributing factors include underdeveloped cognitive control, heightened sensitivity to rewards, and increased sensitivity to peer behavior (Ciranka and van den Bos 2021; Sercombe 2014; Steinberg and Monahan 2007; Willoughby et al. 2013). These same drivers may also shape rule‐breaking behavior. In this paper, we examine how adolescents' rule‐breaking is shaped by peer influence and ambiguous situations that create uncertainty about what to do.

In many everyday situations, the applicability of rules is clear and unambiguous (do not talk over the teacher, wait when a traffic light is red). In many others, cues from the environment make it less clear what to do (Li et al. 2024; Meyer et al. 2010; Mischel 1977). Should you go to a class when attendance is “strongly recommended” but not obligatory? Is it allowed to walk through a yellow traffic light? Such ambiguous situations are open to interpretation, entailing gray areas in which the distinction between compliance and violation is not clearly defined. Ambiguity thus creates “wiggle room” to interpret the situation in self‐serving ways, and recent research with adults shows that many people exploit this ambiguity by breaking the rule to their own benefit (Molleman et al. 2023). However, to date, it is unclear how adolescents' rule‐breaking is influenced by ambiguity.

One might expect that for younger adolescents in particular, ambiguity might increase rule‐breaking when compliance requires suppressing one's impulses to obtain immediate rewards (e.g., Hauser et al. 2015). With age, adolescents' cognitive control matures (Luna 2009) and they become less impulsive (e.g., Blakemore and Robbins 2012; Steinberg et al. 2008), so that ambiguity might affect rule‐compliance less. On the other hand, ambiguity might lead to an increase in compliance among younger adolescents because uncertainty can prompt caution (Braams et al. 2015; Van den Bos and Hertwig 2017). When faced with ambiguous situations where the consequences or expectations of their actions are unclear, younger adolescents may feel unsure about the potential risks of breaking rules, making them more likely to follow rules.

The heightened uncertainty of adolescents has been associated with a strong sensitivity to peer influence (Ciranka and van den Bos 2021; Reiter et al. 2021; Steinberg and Monahan 2007; Laursen and Veenstra 2021; Sumter et al. 2009). Peer influence is particularly important in the context of rule‐breaking (Engel 2023; Gächter et al. 2025; Molleman et al. 2022). When rule‐breaking is contagious, disorder can rapidly spread in groups (Andreoni et al. 2021; Granovetter 1978; Krause et al. 2021), giving rise to collective truancy, school brawls, or protests turning violent. Models show that these processes depend on how individuals' condition their rule‐breaking on that of others, that is, on “descriptive norms” (Bicchieri and Xiao 2009; Granovetter 1978; Gross and Vostroknutov 2022; Gächter et al. 2025). Indeed, people might break a rule only if sufficiently many others break it too (i.e., if their “threshold” is reached; Bicchieri 2008; Bicchieri et al. 2023; Granovetter 1978). A few rule violators may set off a cascade when they are observed by individuals with a low threshold, who then also break the rule, in turn prompting individuals with a higher threshold to also join in.

Empirical studies with adults provide evidence for such conditional rule‐breaking: the more peers break a rule, the more likely people are to follow their example (McGloin and Rowan 2015; Engel 2023). Similarly, observing rule‐following can promote rule‐following (e.g., Gross and De Dreu 2021; Molleman et al. 2022), though recent findings suggest that rule‐breaking examples tend to exert a stronger influence than rule‐following ones (Gächter et al. 2025). Adolescents have been shown to be easily swayed by their peers to break a rule (Molleman et al. 2022; Pasupathi 1999). However, it is unclear how adolescents condition their rule‐breaking on descriptive norms, and how thresholds for rule‐breaking vary between individuals and across adolescence.

Peers may have a direct impact on adolescents' behavior, but may also shape behavior indirectly by influencing what they deem socially acceptable. In adults, descriptive norms are known to influence what people find socially acceptable (“injunctive norms”; Bicchieri 2005; Cialdini and Goldstein 2004; Harms and Skyrms 2008; Lindström et al. 2018), and observing rule‐breaking can undermine a rule's normative appeal (Molleman et al. 2023; Gächter et al. 2025). For example, observing that littering is common can lead people to assume that it is okay to do so. In turn, this can make them more likely to break the rule of “do not litter” themselves (Rowan et al. 2022). Adolescence is an important period for the formation and internalization of such injunctive norms (e.g., Pinho et al. 2021). This internalization often has lasting effects on behavior, as individuals start following rules for intrinsic reasons (as opposed to external pressures like social influence or the threat of punishment; Davis et al. 2018; Gavrilets and Richerson 2017; Gintis 2003). Yet, it remains unclear how observing rule violations or rule adherence shapes adolescents' injunctive norms.

We report on incentivized tasks comparing younger adolescents (11−15 year‐olds; *n* = 296) to older adolescents (16−19 year‐olds; *n* = 333) to understand how situational ambiguity and peer behavior impact adolescent rule‐breaking, and whether adolescents' injunctive norms change in response to observing peer behavior. Our study is organized in three parts.

In part 1, we investigate the extent to which adolescent rule‐breaking depends on situational ambiguity. We hypothesize that situational ambiguity increases rule‐breaking (h1a). We explore the age effects under ambiguity (h1b), and expect that younger adolescents change their rule‐compliance more than older adolescents when the situation is ambiguous (h1c). Since there are arguments for expecting both an increase and a decrease in rule‐compliance under ambiguity among younger adolescents, we refrain from predicting the direction of the effect.

In part 2, we investigate the extent to which adolescent rule‐breaking depends on whether others break the rule. We expect that adolescents' rule‐breaking increases if more of their peers do so too (h2a). Furthermore, we explore age effects on overall compliance (h2b), and we expect that younger adolescents show stronger conditionality on others that break the rule than older adolescents (h2c).

In part 3, we explore whether observing peer behavior influences adolescents' injunctive norms, that is, how socially appropriate they think it is to break the rule. We hypothesize that seeing bad examples (i.e., peers breaking the rule) will make rule‐breaking more socially appropriate. Observing good examples (i.e., peer following the rule) does not change the social appropriateness of rule‐breaking or compliance (h3a). We explore the age effect on injunctive norms (h3b). Furthermore, in line with the diminishing effect of peer influence with age, we expect that the influence of observing peer behavior on injunctive norms is lower for older adolescents (h3c).

Our study aims to elucidate how adolescents' rule‐breaking is shaped by situational ambiguity (part 1) and peer behavior (part 2), and shed light on how peer behavior shapes adolescents' injunctive norms (part 3). We aim to get more insight into the determinants of rule‐breaking across adolescence by comparing younger adolescents to older adolescents. Our contribution lies in providing a nuanced understanding of the social and contextual factors that influence adolescents' adherence to or deviation from rules. This understanding can inform targeted prevention and interventions to encourage rule‐compliance among adolescents.

In our exploratory analyses, we examined whether adolescents show consistent rule‐breaking behavior across tasks (i.e., whether adolescents (younger or older) are sensitive to both ambiguity and peer influence). Furthermore, to validate our experimental results, we related participant behavior to their responses in questionnaires on impulsivity (part 1) and resistance to peer influence (part 2). We also explored how peer effects in rule‐breaking differ between boys and girls. In the discussion section, we compare our results on rule‐breaking in adolescents to recent findings with adult samples.

## 2 Methods

### 2.1 Participants

We recruited 629 Dutch participants (342 [54.4%] female, 11 other) from seven high schools in the Netherlands. To ensure a large age range across adolescence—and thus allow for detecting any differences across adolescence—we targeted students from the first and second year, and from the fifth and sixth year of high school. This led to two age cohorts: (i) younger adolescents ranging from 11 to 15 years of age (*N* = 296, M_age_ = 12.66, SD = 0.80), and (ii) older adolescents ranging from 16 to 19 years of age (*N* = 333, M_age_ = 16.81, SD = 0.79). Note that age heterogeneity within cohorts may reflect students repeating a year or moving between secondary education tracks of differing academic levels.

### 2.2 Procedure

Participants were tested in their own classrooms of 18−45 students, while sitting in an exam‐style setting with spaced seating. At the beginning of each session, the researchers briefly introduced the procedure, experimental tasks, and incentivization (see Supporting Information, Section 5 for the instruction protocol). After this introduction, participants received a participant number and a tablet computer. All participants gave informed consent before starting the tasks. For participants under the age of 16, parents or other caregivers also gave informed consent. Participants were encouraged to ask questions if anything was unclear or when they ran into any difficulties.

To incentivize behavior, participants could earn lottery tickets for a €20 gift voucher that was raffled off after each session. Participants were informed that the number of lottery tickets they could earn depended on their decisions in the tasks. The more tickets they earned, the higher their chances to receive the gift voucher. The voucher could be redeemed on a popular Dutch web shop (Bol.com) offering a broad range of products that appeal to all age groups, from books and games to electronics and fashion. The flexibility to choose items aligned with individual interests helped make the value of the voucher comparable across age cohorts. High schools received €5 for every participating student.

The study was approved by the Ethics Committee of the Faculty of Social and Behavioural Sciences, University of Amsterdam (case number FMG‐2826_2023). The hypotheses, experimental setup, and analyses were registered at https://aspredicted.org/ZX5_D8Q. The registration was done after data collection, but before the authors accessed the data.

### 2.3 Materials

#### 2.3.1 Basic Task

**Figure 1:**
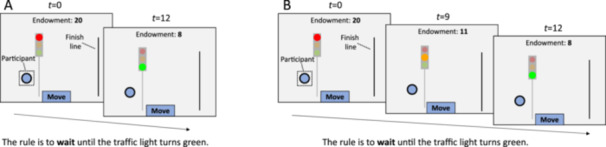
Traffic light task measuring rule‐breaking. Participants controlled a circle that they had to move over the finish line. They were told that the rule is to wait at the traffic light until it turns green. Their initial endowment of 20 lottery tickets was reduced by one ticket each second until they reached the finish line. (A) In the condition with low ambiguity the traffic light turned from red to green after 12 s. (B) In the condition with high ambiguity the traffic light turned from red to yellow after 9 s, before turning green after 12 s. For both conditions, the same rule was stated to participants: wait until the traffic light turns green.

All three parts of our study involved a rule‐following task adapted from Gächter et al. (2025), which was in turn based on the task developed by Kimbrough and Vostroknutov (2016). In this task, participants had to move an animated circle across the screen and over a finish line (Figure 1). As soon as they clicked a “Move” button, the circle approached a red traffic light and stopped to wait. Participants started with an endowment of 20 lottery tickets, but each second in the task, this endowment was reduced by one ticket until they reached the finish line. Participants were thus incentivized to finish the task as soon as possible. However, before starting the task, participants were told that “the rule is to wait until the traffic light turns green,” which happened after 12 s. So, following the rule was against a participant's self‐interest.

We used a within‐subjects design. Throughout the session, participants completed three parts based on this baseline task. Part 1 involved two versions of the task measuring how rule‐breaking depended on situational ambiguity. Part 2 included the behavior of peers. Part 3 measured how peer behavior shaped injunctive norms. The Supporting Information, section 6, shows full instructions for each of the tasks, translated from the Dutch original.

#### 2.3.2 Part 1: Situational Ambiguity Shaping Rule‐Breaking

To measure how situational ambiguity shapes adolescents' rule‐breaking, we administered two versions of the traffic light task, which varied in the degree of ambiguity in the situation. In both versions, participants were instructed that “the rule is to wait until the traffic light turns green.” Participants first completed the version with low ambiguity. They operated the circle and moved it towards the red traffic light, which turned from red to green after 12 s (Figure 1A).

Later on, participants completed the version with high ambiguity. The setup was the same, except that the traffic light first turned from red to yellow after 9 s, before turning green after 12 s (Figure 1B). A red traffic light serves as a classic example of a “strong situation,” clearly indicating the most appropriate action to take (Meyer et al. 2010; Mischel 1977). In contrast, a yellow light provides some room for individuals to interpret the situation in a way that suits them, potentially leading them to bend the rule of waiting for green. In both versions of the task, we coded “waiting for green” (so moving after 12 s) as rule‐following, and “not waiting for green” (i.e., crossing at a red or yellow light) as rule‐breaking.

#### 2.3.3 Part 2: Peer Behavior Shaping Rule‐Breaking

To measure how the behavior of peers shapes adolescents' rule‐breaking, we presented participants with five scenarios which varied in the proportion of previous participants following or breaking the rule. For each situation, participants had to indicate whether they would comply with the rule (i.e., wait until the traffic light turned green) or break the rule (i.e., move before the traffic light turned green). Scenarios varied in the number of peers, out of 100, who broke the rule, presented as deciles (10, 30, 50, 70, or 90).

The five responses of each participant yielded a “strategy profile” indicating whether—and if so, how—they condition their rule‐breaking on the choices of others. This “strategy method” approach (Selten 1967) is a standard approach in cooperation research that is known to yield responses similar to those in actual interactions (Fischbacher et al. 2012). Recently, this method has been successfully deployed in studies on rule‐following (Desmet and Engel 2021; Engel 2023; Gächter et al. 2025). As is standard in this approach, we made the choices incentive‐compatible by using real decisions (in this case, choices in the low ambiguity condition of part 1) to determine the actual proportion of rule‐breaking among peers, and which of the five choices was used to calculate participants' payoffs for part 2.

#### 2.3.4 Part 3: Peer Behavior Shaping Injunctive Norms

To measure how peer behavior shapes adolescents' injunctive norms, we used a method allowing for incentivized elicitation of what people deem socially appropriate (adapted from Krupka and Weber 2013). Participants were asked to rate the “social appropriateness” of rule‐following and rule‐violation in the traffic light task on a scale from 1 to 4 (translated from Dutch: 1 = totally not okay, 2 = not okay, 3 = somewhat okay, 4 = totally okay). After submitting their initial ratings, participants saw the behavior of six peers and were asked to rate the social appropriateness of rule‐following and rule‐violation again. Participants were randomly allocated to one of two conditions, which varied in the behavior of the peers (i.e., setting different descriptive norms). In the “good example” condition, all six peers complied with the rule. In the “bad example” condition, all six peers broke the rule.

After a session was over, participants were randomly paired, and for each pair of participants, we randomly selected one of the four decisions (before or after observing social information, rating for compliance of violation). If the ratings in the pair matched, both participants earned five additional lottery tickets. Participants were informed about this procedure before they started. This incentivization method ensures that participants report how socially appropriate they thought that most people perceived an act of rule‐following or rule‐breaking (rather than their personal opinions about how socially appropriate or inappropriate they perceived rule‐following).

#### 2.3.5 Post‐Experimental Questionnaires

At the end of the test battery, we included two questionnaires to validate our behavioral results. First, we administered the impulsive behavior short scale‐8 (I‐8; *α* = 0.75; Groskurth et al. 2022). Second, we used a simplified version of the Resistance to Peer Influence questionnaire (RPI; *α* = 0.76; Steinberg and Monahan 2007). This simplification was done by converting the original RPI scale into 10 single statements (following Xiao et al. (2023) who showed that this version was reliable and easy to understand for participants). We also collected demographic information (i.e., age and gender).

### 2.4 Statistical Analysis

#### 2.4.1 Confirmatory Analyses

All main analyses were registered at https://aspredicted.org/ZX5_D8Q. For part 1 (situational ambiguity), we used a logistic generalized linear mixed model (GLMM) with rule‐breaking as the dependent variable (comply = 0, break = 1), situational ambiguity (low = 0, high = 1) and age (younger = 0, older = 1) as independent variables, as well as their interaction, and with “participant” as random intercept (h1c). We performed two separate logistic GLMMs for the effect of (i) ambiguity on rule‐breaking and (ii) the effect of age on rule‐breaking, again with participants as random intercept (h1a, h1b).

For part 2 (peer effects), we performed a logistic GLMM with rule‐breaking as dependent variable and the descriptive norm (i.e., how many others broke the rule, coded as a continuous variable with values 10, 30, 50, 70, 90), age (younger/older; dummy‐coded) as independent variables, as well as their interaction, and with participant as random intercept (h2c). In addition, we ran two separate logistic GLMMs to test how rule‐compliance is shaped by (i) the descriptive norm of rule‐compliance and (ii) age group (younger/older; h2a, h2b).

For part 3 (injunctive norms), we ran separate analyses for social appropriateness ratings of (i) violation and (ii) compliance. We ran linear regressions with difference scores in social appropriateness ratings (*after* minus *before* exposure to good or bad examples) as the dependent variable, and the treatment (observing good or bad examples) and age (younger/older; dummy‐coded), and their interaction as independent variables (h3c). In addition, we ran an additional regression analysis to test how difference scores are affected by social information (h3a) and age group (younger/older; h3b).

#### 2.4.2 Explorative Analyses

To explore behavioral consistency across parts 1 and 2, we divided participants into three types (consistent rule‐followers, conditional rule‐breakers, and consistent rule‐breakers). In this exploratory analysis, we quantified consistency with Cohen's κ. In this analysis, we first removed participants who, in part 1, broke the rule under low ambiguity and followed the rule under high ambiguity (*N* = 49). We also removed participants who, in part 2, switched multiple times between rule‐following and rule‐breaking as the percentage of peers who broke the rule increased (*N* = 19, with *N* = 3 overlap with exclusions from part 1) and participants with inverted patterns (starting with rule‐breaking when few peers break the rule, and changing to rule‐following when more peers break the rule; *N* = 17, no overlap with the exclusions from part 1). This leaves us with a sample of *N* = 551 participants: See Supporting Information, Section 3 for details.

We identified the following “types”: (i) *consistent rule‐followers*, who complied with the rule regardless of ambiguity or peer behavior; (ii) *conditional rule‐breakers*, who only broke the rule when ambiguity was high, or who switched from complying to breaking the rule when a certain portion of others also breaks the rule; and (iii) *consistent rule‐breakers*, who always broke the rule regardless of ambiguity or peer behavior.

Furthermore, to explore the effect of gender on rule‐breaking we performed a logistic GLMM with rule‐breaking as dependent variable and the descriptive norm (i.e., how many others broke the rule, coded as a continuous variable with values 10, 30, 50, 70, 90), gender (dummy‐coded) as independent variables, as well as their interaction, and with participant as random intercept. Finally, we explored behavioral consistency across tasks and correlated participants' impulsivity scores and RPI with rule‐breaking.

## 3 Results

In this section we will first discuss the results of the confirmatory analyses of the three parts: situational ambiguity, peer behavior, and injunctive norms. Full model results are presented in the Supporting Information S1: Tables S1−S12. After presenting our confirmatory analyses, we will explore how patterns of rule‐breaking correlate with gender, RPI, and impulsivity.

### 3.1 Part 1: Ambiguity Increases Adolescents' Rule‐Breaking, but This Does Not Differ by Age Group

**Figure 2:**
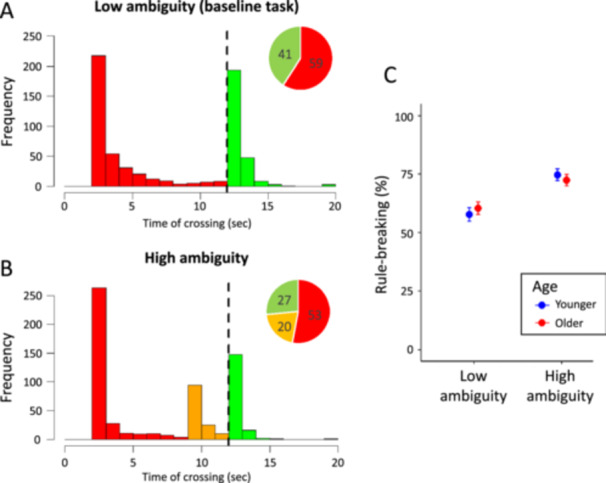
(A and B) Frequencies of time of moving past the traffic light. Bars show participants' timing of their move from the traffic light to the finish line. The colors represent the color of the traffic light. The light turned yellow after 9 s (in the high‐ambiguity version) and green after 12 s (in both task versions). The pie charts in the insets show the percentages of participants moving on green, yellow, and red. (C) Percentage of rule‐breaking in the tasks with low and high ambiguity, broken down by age group.

When ambiguity was low, 59.1% of participants broke the rule by directly moving their circle across the finish line upon reaching the red traffic light (Figure 2A). Most of the remaining 40.9% of participants moved directly after the traffic light turned green. When ambiguity was high, the portion of participants breaking the rule increased to 73.4% (Figure 2B), supporting hypothesis 1a (B = 1.00, *p* < 0.001, OR = 2.73, 95% CI [1.98, 3.75]; Supporting Information S1: Table S1). This increased rule‐breaking was mainly due to 20% of participants moving when the traffic light turned yellow (i.e., when the situation was ambiguous). Age did not significantly impact rule‐breaking (h1b; B = 0.03, *p* = 0.891, OR = 1.03, 95% CI [0.71, 1.49]; Supporting Information S1: Table S2).

We did not observe a significant interaction between age and ambiguity (Figure 2C; B = −0.33, *p* = 0.283, OR = 0.72, 95% CI [0.40, 1.31]; Supporting Information S1: Table S3). When ambiguity was low, the rule was broken by 58% of younger adolescents and 60% of older adolescents. In the high‐ambiguity task, these percentages were 75% and 72%, respectively. Results for the interaction effect stay the same if we include age as a continuous variable in the analysis (B = −0.07, *p* = 0.331, OR = 0.94, 95% CI [0.82, 1.07]). These results are also robust to mixed‐effects models specifying participants nested within classrooms as random effects (Supporting Information S1: Tables S19–S21).

As predicted, the results of part 1 show that situational ambiguity substantially increases rule‐breaking. Unexpectedly, however, this effect is similar for younger and older adolescents.

### 3.2 Part 2: Peer Effects on Rule‐Breaking Are Strong

**Figure 3:**
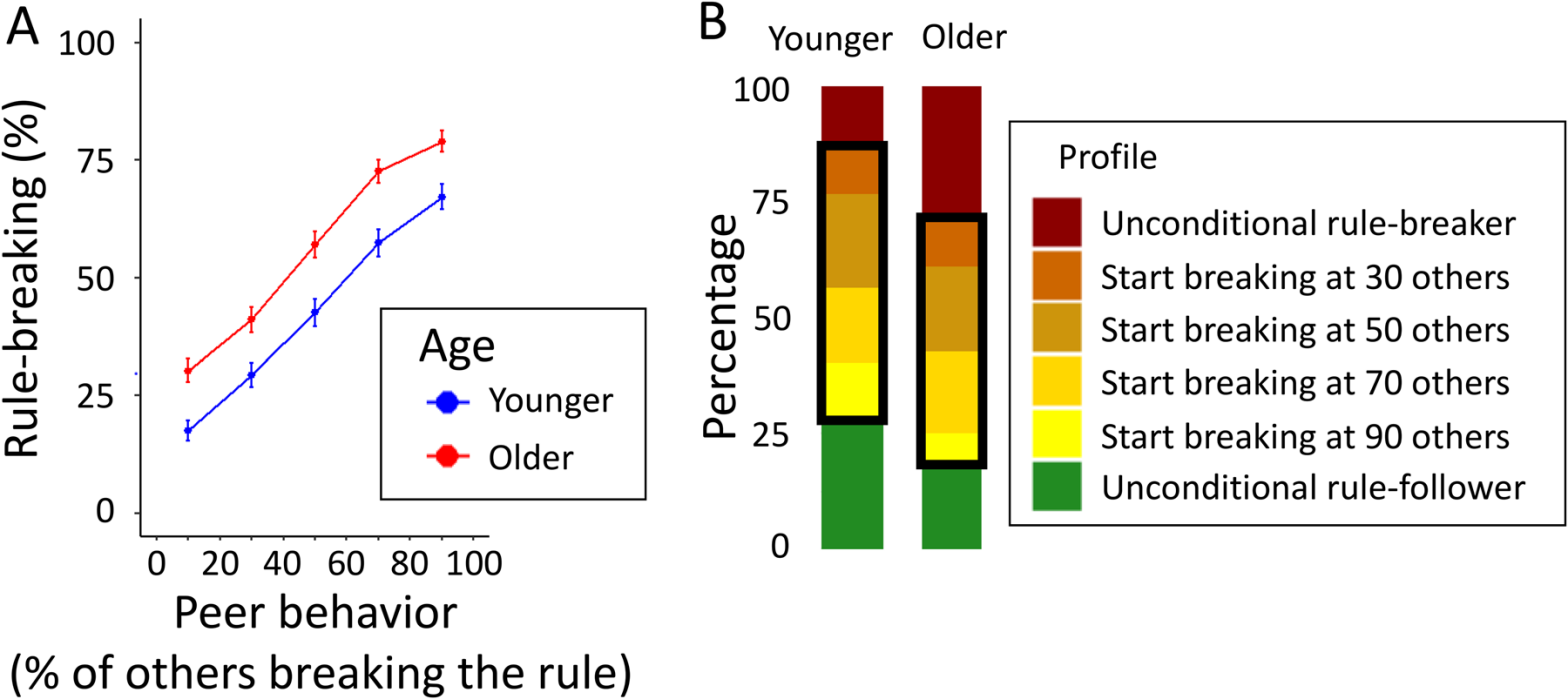
(A) Rule‐breaking as a function of the percentage of others breaking the rule. The dots show proportions (±1 SEM) for each decile, broken down by age group. (B) Distribution of response profiles across age excluding inconsistent responses (*N* = 19 of which 14 younger adolescents and five older adolescents). The black boxes highlight conditional strategies, with colors indicating participants' thresholds for breaking the rule (i.e., when at least 30, 50, 70, or 90 out of 100 others broke the rule).

Supporting hypothesis 2a, we found that rule‐breaking increased when more peers broke the rule (Figure 3A; B = 0.07, *p* < 0.001, OR = 1.07, 95% CI [1.06, 1.08]; Supporting Information S1: Table S4). For both younger and older adolescents, rule‐breaking increased with roughly 50 percentage points (pp) when peers were mostly following versus breaking the rule (from 17.6% to 67.2%, and from 30.3% to 79.0%, respectively).

The distribution of response profiles can be found in Figure 3B, broken down by age group. Among the younger adolescents, 13.5% are unconditional rule‐breakers, breaking the rule regardless of what others do. By contrast, 28.0% are unconditional rule‐followers. Compared to younger adolescents, older adolescents were more often rule‐breakers (28.7%), and less often rule‐followers (18.3%).

A large proportion of participants (younger adolescents: 55.5%; older adolescents: 53.0%) had conditional strategies (Figure 3B; black boxes). These participants complied with the rule until a threshold was reached, with most of them switching to breaking the rule when 50 or 70 out of 100 others would also break the rule (Figure 3B; dark yellow stacked bars). In this analysis, we excluded 19 participants with inconsistent response patterns (i.e., who switched multiple times between rule‐following and rule‐breaking as the fraction of others breaking the rule increased).

Overall, older adolescents were more likely to break the rule (h2b; B = 0.89, *p* < 0.001, OR = 2.44, 95% CI [1.71, 3.59]; red dots in Figure 3A; Supporting Information S1: Table S5). Contrary to our hypothesis (h2c), we did not find a significant interaction between age and peer behavior on rule‐breaking (B = 0.01, *p *= 0.132, OR = 1.01, 95% CI [1.00, 1.02]; Supporting Information S1: Table S6), also not when including age as a continuous variable in the analysis (B = 0.00, *p* = 0.216, OR = 1.00, 95% CI [1.00, 1.00]). These results are robust to mixed‐effects models specifying participants nested within classrooms as random effects (Supporting Information S1: Tables S22–S24).

Taken together, the results of part 2 show that adolescents' rule‐breaking strongly depends on peer behavior, and many adolescents use conditional strategies. However, the degree of conditionality does not differ between younger and older adolescents. Overall, older adolescents broke the rule more often than younger adolescents.

### 3.3 Part 3: Older Adolescents' Injunctive Norms Are More Sensitive to Peer Behavior

**Figure 4:**
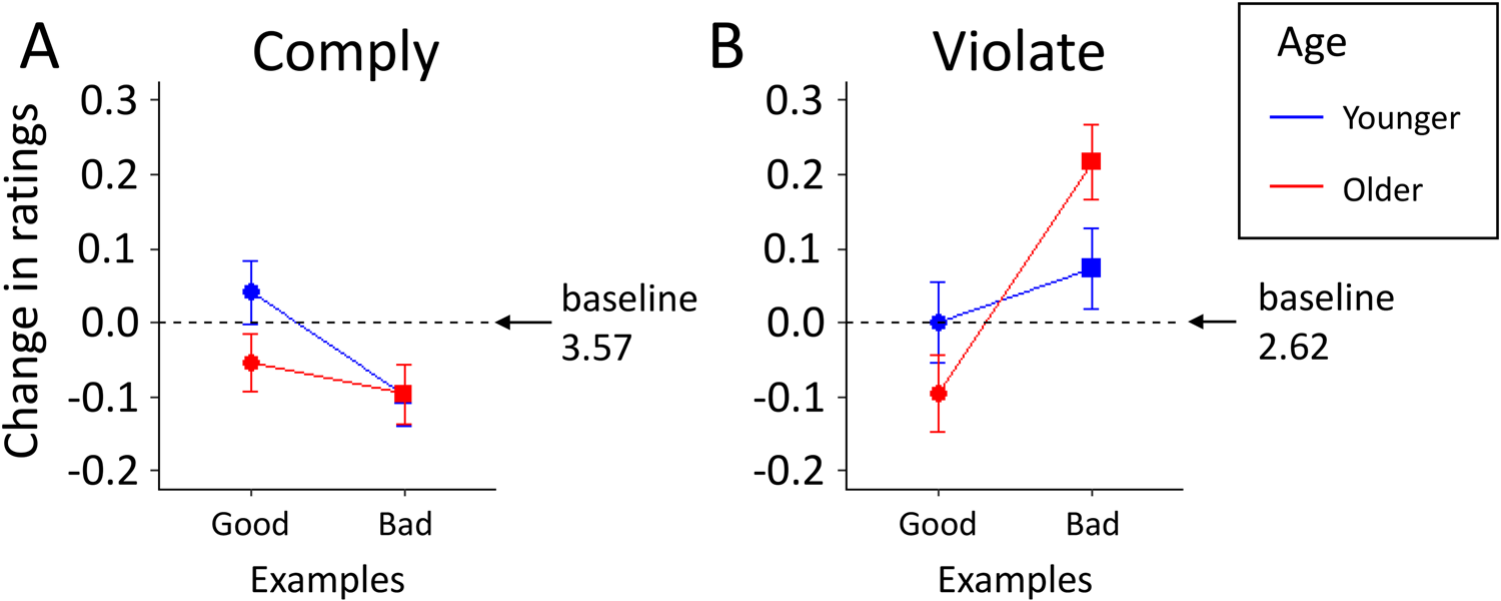
(A and B) The impact of peer behavior on injunctive norms. Panels show changes in social appropriateness ratings for compliance (A) and violation (B) after seeing good or bad examples, broken down by age group. The ratings were between 1 = “totally not OK” and 4 = “totally OK.” Symbols show mean changes ±1 SEM. See Supporting Information S1: Figure S1 for full distributions of responses for each case.

Figure 4 shows social appropriateness ratings for rule compliance and violation before and after observing peers either following or breaking the rule (i.e., providing good or bad examples). Before seeing the examples, ratings were significantly higher for rule‐compliance (M = 3.57, SD = 0.71 on a 1−4 scale) than for rule‐violation (M = 2.62, SD = 0.97; paired‐*t*‐test: *t*[628] = 19.85, *p* < 0.001; Figure 4A, B, horizontal lines indicate these baselines).

Observing peer behavior did not chance ratings for compliance much (Figure 4A; Supporting Information S1: Table S7). Ratings for violation slightly decreased after observing compliance (Figure 4B, dot), and increased after observing violations (Figure 4B; Supporting Information S1: Tables S8−10). This suggests that bad examples reduce a rule's normative appeal. Contrary to predictions, compared to younger adolescents, older adolescents' injunctive norms were slightly more sensitive to observing peer behavior, particularly when rating rule violations (Supporting Information S1: Tables S11,12).

Only a subset of participants changed their ratings after observing examples. Ratings for compliance were updated by 14% and 18% of participants after observing good or bad examples, respectively. Ratings for violations were updated by 26% and 32% of participants after observing good or bad examples, respectively. This indicates that for a large majority of participants, injunctive norms did not change upon observing good or bad behavior by others.

### 3.4 Exploratory Analyses

#### 3.4.1 Behavioral Consistency Across Tasks

Applying our exclusion criteria (see Methods) left us with 551 participants for this analysis (see Supporting Information for details). Cross‐tabulation of the behavioral types for the tasks in parts 1 and 2 shows that 66 participants (12.0%) are consistent rule‐followers, 110 participants (20.0%) are consistent rule‐breakers, and 14.7% of participants are conditional rule‐breakers in both parts (Table 1, diagonal). This means that 46.7% of participants showed consistent behavior across parts of our study (weighted Cohen's κ = 0.43; if excluded cases are included, Cohen's κ = 0.41). This suggests that adolescents whose rule‐breaking is sensitive to ambiguity also tend to be more sensitive to peer behavior. Moreover, and perhaps unsurprisingly, consistent rule‐breakers in either part 1 or part 2 think violation is less socially inappropriate than people who are conditional rule‐breakers or consistent rule‐followers in these tasks (see Supporting Information Section 3).

**Table 1:** Distributions of participants across behavioral “types.” Based on parts 1 and 2, participants were classified as consistent rule‐followers, conditional rule‐breakers (making rule‐breaking depend on ambiguity and peer behavior), and consistent rule‐breakers.

		Part 2: Peer behavior		
		Unconditional rule‐followers	Conditional rule‐breakers	Unconditional rule‐breakers
**Part 1: ambiguity**	**Unconditional rule‐followers**	66 (12.0%)	43 (7.8%)	6 (1.1%)
	**Conditional rule‐breakers**	34 (6.2%)	81 (14.7%)	12 (2.2%)
	**Unconditional rule‐breakers**	23 (4.2%)	176 (31.9%)	110 (20.0%)

#### 3.4.2 Gender Differences in Peer Effects in Rule‐Breaking

**Figure 5:**
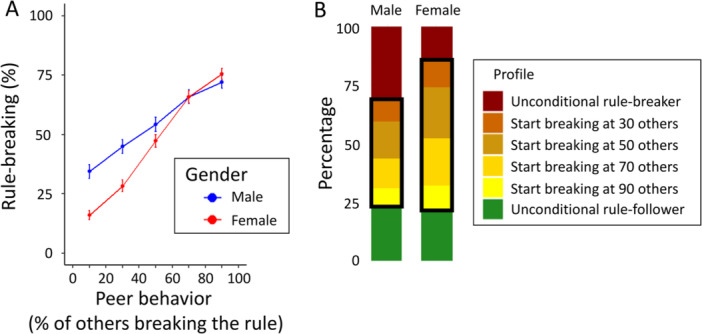
(A) Rule‐breaking as a function of the proportion of others breaking the rule, broken down by gender. Dots and whiskers show means ±1 SEM for each decile. (B) Distribution of response profiles by gender. Profiles in the black boxes indicate conditional types.

In part 2, we observed pronounced gender differences in rule‐breaking (Figure 5). Overall, boys were more likely to break the rule than girls (Figure 5A; B = −0.53, *p* = 0.004, OR = 0.59, 95% CI [0.41, 0.84]; Supporting Information S1: Table S15); especially when most others followed the rule (interaction gender x peer behavior: B = 0.04, *p* < 0.001, OR = 1.04, 95% CI [1.03, 1.05]; Supporting Information S1: Table S16). When most peers broke the rule, both boys and girls were very likely to break the rule, with no differences between the genders.

Participants' response profiles reveal that boys were more likely to be unconditional rule‐breakers than girls (31.4% vs. 14.3%; *χ* ^2^ [1] = 24.31, *p *< 0.001, Figure 5B). Girls are substantially more likely to use a conditional strategy than boys (63.9% vs. 45.1%; *χ* ^2^ [1] = 20.394 *p* < 0.001). Interestingly, in part 1 we do not find any gender effects in rule‐breaking (Supporting Information S1: Table S13−S16).

#### 3.4.3 Impulsivity Predicts Rule‐Breaking, RPI Predicts Conditionality

Impulsivity correlated positively with rule‐breaking in part 1 when ambiguity was low (*r*[628] = 0.24, *p* < 0.001) and when it was high (*r*[628] = 0.16, *p* < 0.001). This indicates that rule‐breaking in the behavioral tasks may be partly explained by impulsiveness (see Supporting Information S1: Table S17, S18 for details). Furthermore, participants who used a conditional strategy in part 2 had somewhat lower RPI (M = 3.00, SD = 0.35) than those who unconditionally broke or followed the rule (M = 3.06, SD = 0.42; *t*[546] = 2.04, *p* = 0.042). These results provide validation of our behavioral results.

## 4 Discussion

We examined determinants of adolescents' rule‐breaking in incentivized experimental tasks, organized in three parts. Part 1 showed that situational ambiguity substantially increases rule‐breaking, and does so equally across adolescence. Part 2 showed that adolescents' rule‐breaking is strongly influenced by peer behavior: rule‐breaking increases when more others break the rule as well. The strength of peer effects did not differ by age group. Interestingly, exploratory results showed that girls were substantially more sensitive to peer influence than boys, many of whom broke the rule regardless of others' behavior. Overall, older adolescents broke the rule more often than younger adolescents. Part 3 showed that observing peer behavior can lead to slight changes in injunctive norms, particularly among older adolescents.

In part 1, 59% of adolescents broke the rule under low ambiguity (Figure 2), a higher rate than American adults completing the same task online (42%; Molleman et al. 2023) and German adolescents in the lab (47%; Molleman et al. 2022). The presence of peers in our classroom setting may have increased rule‐breaking (e.g., Gardner and Steinberg 2005) while stricter adherence to rules in lab and online settings may have reduced rule‐breaking in other studies (Zizzo 2010). Although methodological differences limit direct comparisons between Dutch and German adolescents (and American adults), the findings point to possible cross‐cultural differences in rule‐breaking. Future research applying standardized methods across wider age groups could clarify how cultural values influence the development of rule‐following, e.g., by emphasizing intrinsic respect for rules or conformity to social norms (Gächter et al. 2025; Gächter and Schulz 2016; Surachita 2022).

Part 1 showed that rule‐breaking increased from 59% under low ambiguity to 73% when ambiguity was high (Figure 2). This result indicates that, as expected, ambiguity substantially promotes rule‐breaking in adolescence. This 14 pp rise is similar to the 19pp increase observed in adults (Molleman et al. 2023), where rule‐breaking rose from 42% to 61%. Thus, while adolescents are sensitive to ambiguity, this sensitivity does not appear to be greater than in adults. Overall, the results of part 1 indicate that although rule‐breaking is high among adolescents, it can be curbed when the situation leaves little room to interpret a rule to one's own advantage. At the same time, when situational ambiguity is high, we observe a minor reduction of participants moving on red (6 pp; Figure 2A,B), suggesting that ambiguity can make people “bend” the rule (i.e., moving on yellow) when they would otherwise break it. While in the short term this might slightly reduce the overall extent of rule breaking, observing others bending the rule may prompt further rule breaking (cf. Figure 3; Molleman et al. 2023).

Indeed, part 2 showed that adolescents' rule‐breaking is strongly influenced by peer behavior (Figure 3). Rule‐breaking increased by about 50 pp when the proportion of peers breaking the rule rose from 10% to 90% (Figure 3A), a larger increase than seen in adults (35 pp; Gächter et al. 2025). Similarly, a substantiation portion (54%) of adolescents used conditional strategies, higher than in recent adult studies (28% in Engel 2023; 31% in Gächter et al. 2025). This underscores adolescents' heightened sensitivity to peer influence, likely driven by their strong desire for acceptance (Chierchia et al. 2020; Laursen and Veenstra 2021; Pasupathi 1999; Wright et al. 1986). The pronounced conditionality suggests that rule‐breaking dynamics in adolescent groups may be more volatile than in adults, with disorder potentially spreading rapidly once a tipping point is reached (Granovetter 1978; Krause et al. 2021; Andreoni et al. 2021).

Exploratory analysis shows that this conditionality was especially strong for girls (Figure 5A). Their responses suggest that peer influence can sway them in both undesirable and desirable directions alike. This pattern is different for boys, who broke the rule more often than girls (Figure 5A). This result is in line with recent experimental findings (Bicchieri et al. 2025; Gächter et al. 2025), and with studies showing that boys engage in higher risk‐taking and delinquent behavior (e.g., Byrnes et al. 1999; Mears et al. 1998; Moffitt et al. 2001). This difference might be partly explained by traditional gender norms tolerating or even glorifying rebellious behavior in boys (e.g., Connell 2005). Breaking rules—even when most others are following them (cf. Figure 5A)—may be endorsed as expressions of male autonomy, while similar behaviors in girls might be viewed negatively.

Furthermore, the exploratory analysis on behavioral consistency across tasks (Table 1) revealed that many participants consistently broke the rule when deciding alone (part 1) but followed the rule when sufficiently many others did so too (part 2). It remains an open question to what extent these patterns might reflect behavioral types, and whether they are associated with common cognitive or personality traits such as self‐control, social sensitivity, or emotional stability (Cubillos‐Pinilla and Emmerling 2022; Tate et al. 2022). Regardless, our results highlight that observing the behavior of others can prompt rule breakers to start following the rule (Gross and De Dreu 2021; Molleman et al. 2022), and add to mounting evidence that social influence has the potential to change adolescents' behavior for the good (Ahmed et al. 2020; Chung et al. 2020; Van Hoorn et al. 2016).

Part 3 suggests that peer influence on injunctive norms increases with adolescents' age (Figure 4). One explanation for this might be that for young adolescents, injunctive norms are mainly influenced by other sources, for example, their parents or teachers (Lew‐Levy et al. 2023), while for older adolescents the focus has shifted to classmates and friends (Albert and Steinberg 2011; Crone and Dahl 2012; Molleman et al. 2019; Pinho et al. 2021). Although the effects in our experiments were small, our results may provide an interesting starting point for research on how different social sources shape the development of injunctive norms during adolescence.

Interestingly, younger and older adolescents did not differ in rule‐breaking in the behavioral task (part 1; Figure 2), but older adolescents showed substantially more rule‐breaking in the presence of peers (Figure 3A). The latter result aligns with the observation that risky and unruly behavior peaks in late adolescence (Icenogle and Cauffman 2021; Shulman et al. 2013). Our results suggest that older adolescents are particularly sensitive to the mere presence of a social context in which they observe peer behavior, whether positive or negative. Since much adolescent rule‐breaking occurs in social settings, future research could clarify the extent to which this behavior is influenced by the mere presence of peers versus the specific behaviors peers exhibit.

Our study tested how adolescents' rule‐breaking is impacted by situational ambiguity, in which the yellow light provided a contextual cue aiming to increase uncertainty about what to do (Mischel 1977; Meyer et al. 2010), and to create room for participants to interpret the situation in a self‐serving way. In everyday life, rule‐breaking may be affected by various kinds of ambiguity, such as rule ambiguity, moral ambiguity, or linguistic ambiguity. Our experimental task may serve as a template to study how these different kinds of ambiguity affect rule‐breaking, and how their effects may differ across development. Future studies may also address to what extent our results are specific to situations involving traffic lights. Although in our experiment we explicitly stated that the rule was to wait on green, participants' reactions might be shaped by real‐world experiences that moving on yellow is permitted (see Alekseev et al. 2017 for further discussion of contextualized experiments). Experiments based on abstract rule‐following paradigms (e.g., Kimbrough and Vostroknutov 2018; Gäechter et al. 2025) may help test to what extent our results depend on the decision context and our operationalization of situational ambiguity.

Following earlier studies in Dutch high schools (e.g., Pinho et al. 2021; Gradassi et al. 2023), we used a raffle‐based incentive, where one student per classroom could win a gift voucher. While logistically convenient, this zero‐sum setup may have encouraged rule‐breaking among participants motivated to outcompete their peers. Although we did not measure competitiveness, it is conceivable that it may be stronger in older adolescents and boys (see Dreber et al. 2014 for a discussion). This could partly explain the higher rates of rule‐breaking we observe in these groups (see Figures 3A and 5). Younger participants might have perceived the voucher as more valuable due to having fewer alternative sources of income or spending power than older participants. However, this is unlikely to explain the age pattern we observed, especially given that the voucher could be used for products appealing across age groups (see Methods), and that rule‐breaking was higher among older adolescents.

In addition, all participants completed the tasks in a fixed order, beginning with the simplest (low‐ambiguity behavioral task) and ending with the more complex ones (strategy method and high‐ambiguity task) to support comprehension and engagement. While this likely reduced confusion, it may have introduced carryover effects. Recent work using the same traffic‐light paradigm shows that rule‐following tends to increase across repeated decisions (Gächter et al. 2025), suggesting our observed increase in rule‐breaking under high ambiguity may be a conservative estimate. Confirming this conjecture would require a counterbalanced or between‐subjects design.

Taken together, our study shows that ambiguity substantially increases adolescents' rule‐breaking. Compliance may be enhanced by clear communication of expectations and leaving little wiggle room for adolescents to interpret the situation to their own advantage. The strong peer effects on rule‐breaking highlight the key role of social context, which can not only influence adolescents for the ill, but also for the good.

## Author Contributions

**Karlijn Hoyer:** conceptualization, analysis, methodology, project administration, visualization, writing – original draft. **Jelle Sijtsema:** conceptualization, funding acquisition, writing – review and editing. **Christoph Kogler:** conceptualization, funding acquisition, writing – review and editing. **Wouter van den Bos:** funding acquisition, writing – review and editing. **Lucas Molleman:** conceptualization, funding acquisition, methodology, software, supervision, visualization, writing – original draft, writing – review and editing.

## Ethics Statement

All studies reported in this paper have received ethical approval from the Ethics Review Board of the Developmental Psychology department of the University of Amsterdam (Case Number: FMG‐2826_2023).

## Consent

Parental consent was obtained for all participants younger than 16.

## Conflicts of Interest

The authors declare no conflicts of interest.

## Supporting information

Hoyer et al. ‐ SI ‐ Rule Breaking in Aolescence.

## Data Availability

All data and code associated with this paper are publicly available on https://osf.io/jrsg2/?view_only=bb8b68fc7f42451983c837eb37ba8eff.
