# Supplementary material for: Determinants of Rule‐Breaking in Adolescence
Source: J Adolesc. 2025 Aug 12;97(8):2185–97. doi: 10.1002/jad.70031 (PMC12682245; doi:10.1002/jad.70031)
Supplement: Supplementary file 1 — Hoyer et al. ‐ SI ‐ Rule Breaking in Aolescence. [file JAD-97-2185-s001.docx]

Supplementary Information for

**Determinants of rule-breaking in adolescence**

By Karlijn Hoyer, Jelle Sijtsema, Christoph Kogler, Wouter van den Bos,
and Lucas Molleman

**Table of contents**

1. Regression Tables
2. Supplementary Figure: Distribution of responses in part 3
3. Supplementary Methods: Behavioral consistency across tasks
4. Exploratory Analyses: Impulsivity and resistance to peer influence
5. Instruction protocol (translated from Dutch)
6. Full battery of tasks and questionnaires (translated from Dutch)

**1. Regression Tables**

**Table S1**

Logistic generalized linear mixed model (GLMM) with rule-breaking in part 1 as the dependent variable (comply=0, violate=1) and situational ambiguity (low=0, high=1) as independent variable, and with ‘participant’ as random intercept (h1a).

| Predictor | B | SE | OR | 95% CI for OR | p-value |
| --- | --- | --- | --- | --- | --- |
| Fixed effects |  |  |  |  |  |
| Intercept | 0.61 | 0.13 | 1.83 | [1.41, 2.39] | < .001 |
| Ambiguity | 1.00 | 0.16 | 2.73 | [1.98, 3.75] | < .001 |
|  |  |  |  |  |  |
| Random effects |  |  |  |  |  |
| Variance of participant intercept (Level 2) | 3.05 (1.75) |  |  |  |  |

Notes: B = unstandardized coefficient; SE = standard error; OR = odds ratio; CI = confidence interval. standard deviations in parentheses for random effects.

**Table S2**

Logistic generalized linear mixed model (GLMM) with rule-breaking in part 1 as the dependent variable (comply=0, violate=1) and age (younger=0, older=1) as independent variable, and with ‘participant’ as random intercept (h1b).

| Predictor | B | SE | OR | 95% CI for OR | p-value |
| --- | --- | --- | --- | --- | --- |
| Fixed effects |  |  |  |  |  |
| Intercept | 0.99 | 0.15 | 2.68 | [2.00, 3.61] | < .001 |
| Age | 0.03 | 0.19 | 1.03 | [0.71, 1.49] | .891 |
|  |  |  |  |  |  |
| Random effects |  |  |  |  |  |
| Variance of participant intercept (Level 2) | 2.27 (1.51) |  |  |  |  |

Notes: B = unstandardized coefficient; SE = standard error; OR = odds ratio; CI = confidence interval. standard deviations in parentheses for random effects.

**Table S3**

Logistic generalized linear mixed model (GLMM) with rule-breaking in part 1 as the dependent variable (comply=0, violate=1) and ambiguity (0=low, 1=high), age (younger=0, older=1) and the interaction between ambiguity and age as independent variables, and with ‘participant’ as random intercept (h1c).

| Predictor | B | SE | OR | 95% CI for OR | p-value |
| --- | --- | --- | --- | --- | --- |
| Fixed effects |  |  |  |  |  |
| Intercept | 0.51 | 0.19 | 1.67 | [1.16, 2.40] | .006 |
| Ambiguity | 1.18 | 0.23 | 3.24 | [2.06, 5.10] | < .001 |
| Age | 0.18 | 0.25 | 1.20 | [0.73, 1.97] | .467 |
| Ambiguity * Age | -0.33 | 0.30 | 0.72 | [0.40, 1.31] | .283 |
|  |  |  |  |  |  |
| Random effects |  |  |  |  |  |
| Variance of participant intercept (Level 2) | 3.06 (1.75) |  |  |  |  |

Notes: B = unstandardized coefficient; SE = standard error; OR = odds ratio; CI = confidence interval. standard deviations in parentheses for random effects.

**Table S4**

Logistic generalized linear mixed model (GLMM) with rule-breaking in part 2 as the dependent variable (comply=0, violate=1) and peer behavior (% of others breaking the rule as independent variable, and with ‘participant’ as random intercept (h2a).

| Predictor | B | SE | OR | 95% CI for OR | p-value |
| --- | --- | --- | --- | --- | --- |
| Fixed effects |  |  |  |  |  |
| Intercept | -3.47 | 0.23 | 0.03 | [0.02. 0.05] | < .001 |
| Peer behavior | 0.07 | 0.00 | 1.07 | [1.06, 1.08] | < .001 |
|  |  |  |  |  |  |
| Random effects |  |  |  |  |  |
| Variance of participant intercept (Level 2) | 12.59 (3.55) |  |  |  |  |

Notes: B = unstandardized coefficient; SE = standard error; OR = odds ratio; CI = confidence interval. standard deviations in parentheses for random effects.

**Table S5**

Logistic generalized linear mixed model (GLMM) with rule-breaking in part 2 as the dependent variable (comply=0, violate=1) and age (younger=0, older=1) as independent variable, and with ‘participant’ as random intercept (h2b).

| Predictor | B | SE | OR | 95% CI for OR | p-value |
| --- | --- | --- | --- | --- | --- |
| Fixed effects |  |  |  |  |  |
| Intercept | -0.49 | 0.13 | 0.61 | [0.47, 0.80] | < .001 |
| Age | 0.89 | 0.18 | 2.44 | [1.71, 3.49] | < .001 |
|  |  |  |  |  |  |
| Random effects |  |  |  |  |  |
| Variance of participant intercept (Level 2) | 3.71 (1.93) |  |  |  |  |

Notes: B = unstandardized coefficient; SE = standard error; OR = odds ratio; CI = confidence interval. standard deviations in parentheses for random effects.

**Table S6**

Logistic generalized linear mixed model (GLMM) with rule-breaking in part 2 as the dependent variable (comply=0, violate=1) and peer behavior (% of other breaking the rule), age (younger=0, older=1) and the interaction between peer behavior and age as independent variables, and with ‘participant’ as random intercept (h2c).

| Predictor | B | SE | OR | 95% CI for OR | p-value |
| --- | --- | --- | --- | --- | --- |
| Fixed effects |  |  |  |  |  |
| Intercept | -4.10 | 0.33 | 0.02 | [0.01, 0.03] | < .001 |
| Peer behavior | 0.07 | 0.00 | 1.07 | [1.06, 1.08] | < .001 |
| Age | 1.16 | 0.42 | 3.18 | [1.39, 7.26] | .006 |
| Peer behavior * Age | 0.01 | 0.01 | 1.01 | [1.00, 1.02] | .132 |
|  |  |  |  |  |  |
| Random effects |  |  |  |  |  |
| Variance of participant intercept (Level 2) | 12.37 (3.52) |  |  |  |  |

Notes: B = unstandardized coefficient; SE = standard error; OR = odds ratio; CI = confidence interval. standard deviations in parentheses for random effects.

**Table S7**

Linear regression with difference in social appropriateness ratings of compliance as the dependent variable and example type (bad example = 0, good example = 1) as independent variable.

| Predictor | B | SE | p-value |
| --- | --- | --- | --- |
| Intercept | -0.10 | 0.03 | < .001 |
| Example type | 0.09 | 0.04 | .030 |
|  |  |  |  |
| R^2^ | .01 |  |  |
| F-statistic | 4.70 |  | .030 |

Notes: B = unstandardized coefficient; SE = standard error

**Table S8**

Linear regression with difference in social appropriateness ratings of violation as the dependent variable and example type (bad example = 0, good example = 1) as independent variable.

| Predictor | B | SE | p-value |
| --- | --- | --- | --- |
| Intercept | 0.15 | 0.03 | < .001 |
| Example type | -0.20 | 0.05 | < .001 |
|  |  |  |  |
| R^2^ | .02 |  |  |
| F-statistic | 14.16 |  | < .001 |

Notes: B = unstandardized coefficient; SE = standard error

**Table S9**

Linear regression with absolute difference in social appropriateness ratings of compliance as the dependent variable and age (younger = 0, older = 1) as independent variable.

| Predictor | B | SE | p-value |
| --- | --- | --- | --- |
| Intercept | 0.21 | 0.03 | < .001 |
| Age | -0.04 | 0.04 | .349 |
|  |  |  |  |
| R^2^ | .00 |  |  |
| F-statistic | 0.88 |  | .349 |

Notes: B = unstandardized coefficient; SE = standard error

**Table S10**

Linear regression with absolute difference in social appropriateness ratings of violation as the dependent variable and age (younger = 0, older = 1) as independent variable. NB: baseline social appropriateness ratings (before seeing examples) for both compliance and violation (the horizontal dotted lines in Fig. 4 of the main text) were higher for older than for younger adolescents (compliance: M = 3.65, SD = 0.68 vs M = 3.47, SD = 0.71; t(627) = 3.25, p = .001; violation: M = 2.70, SD = 0.95 vs M = 2.52, SD = 1.00; t(627) = 2.43 p = .015).

| Predictor | B | SE | p-value |
| --- | --- | --- | --- |
| Intercept | 0.33 | 0.03 | < .001 |
| Age | 0.02 | 0.05 | .657 |
|  |  |  |  |
| R^2^ | .00 |  |  |
| F-statistic | 0.20 |  | .658 |

Notes: B = unstandardized coefficient; SE = standard error

**Table S11**

Linear regression with difference in social appropriateness ratings of compliance as the dependent variable and, example type (bad example = 0, good example = 1), age (younger = 0, older = 1) and example type * age as independent variables.

| Predictor | B | SE | p-value |
| --- | --- | --- | --- |
| Intercept | -0.10 | 0.04 | .017 |
| Example type | 0.14 | 0.06 | .018 |
| Age | 0.00 | 0.06 | .959 |
| Example type * Age | -0.10 | 0.08 | .228 |
|  |  |  |  |
| R^2^ | .01 |  |  |
| F-statistic | 2.48 |  | .060 |

Notes: B = unstandardized coefficient; SE = standard error

**Table S12**

Linear regression with difference in social appropriate ratings of violation as the dependent variable and, example type (bad example = 0, good example = 1), age (younger = 0, older = 1) and the interaction between example type and age as independent variables.

| Predictor | B | SE | p-value |
| --- | --- | --- | --- |
| Intercept | 0.07 | 0.05 | .178 |
| Example type | -0.07 | 0.08 | .0345 |
| Age | 0.14 | 0.07 | .054 |
| Example type * Age | -0.24 | 0.11 | .024 |
|  |  |  |  |
| R^2^ | .03 |  |  |
| F-statistic | 6.53 |  | < .001 |

Notes: B = unstandardized coefficient; SE = standard error

**Table S13**

Logistic generalized linear mixed model (GLMM) with rule-breaking in part 1 as the dependent variable (comply=0, violate=1) and gender (male=0, female=1) as independent variable, and with ‘participant’ as random intercept.

| Predictor | B | SE | OR | 95% CI for OR | p-value |
| --- | --- | --- | --- | --- | --- |
| Fixed effects |  |  |  |  |  |
| Intercept | 1.11 | 0.16 | 3.05 | [2.23, 4.16] | < .001 |
| Gender | -0.23 | 0.19 | 0.79 | [0.54, 1.15] | .221 |
|  |  |  |  |  |  |
| Random effects |  |  |  |  |  |
| Variance of participant intercept (Level 2) | 2.15 (1.47) |  |  |  |  |

Notes: B = unstandardized coefficient; SE = standard error; OR = odds ratio; CI = confidence interval. standard deviations in parentheses for random effects.

**Table S14**

Logistic generalized linear mixed model (GLMM) with rule-breaking in part 1 as the dependent variable (comply=0, violate=1) and ambiguity (0=low, 1=high), gender (male=0, female=1) and the interaction between ambiguity and gender as independent variables, and with ‘participant’ as random intercept.

| Predictor | B | SE | OR | 95% CI for OR | p-value |
| --- | --- | --- | --- | --- | --- |
| Fixed effects |  |  |  |  |  |
| Intercept | 0.66 | 0.20 | 1.94 | [1.33, 2.85] | < .001 |
| Ambiguity | 1.15 | 0.24 | 3.18 | [1.98, 5.10] | < .001 |
| Gender | -0.13 | 0.25 | 0.87 | [0.53, 1.44] | .602 |
| Ambiguity * Gender | -0.28 | 0.31 | 0.76 | [0.41, 1.38] | .361 |
|  |  |  |  |  |  |
| Random effects |  |  |  |  |  |
| Variance of participant intercept (Level 2) | 2.90 (1.70) |  |  |  |  |

Notes: B = unstandardized coefficient; SE = standard error; OR = odds ratio; CI = confidence interval. standard deviations in parentheses for random effects.

**Table S15**

Logistic generalized linear mixed model (GLMM) with rule-breaking in part 2 as the dependent variable (comply=0, violate=1) and gender (male=0, female=1) as independent variable, and with ‘participant’ as random intercept.

| Predictor | B | SE | OR | 95% CI for OR | p-value |
| --- | --- | --- | --- | --- | --- |
| Fixed effects |  |  |  |  |  |
| Intercept | 0.30 | 0.14 | 1.35 | [1.03, 1.77] | .031 |
| Gender | -0.53 | 0.19 | 0.59 | [0.41, 0.84] | .004 |
|  |  |  |  |  |  |
| Random effects |  |  |  |  |  |
| Variance of participant intercept (Level 2) | 3.82 (1.95) |  |  |  |  |

Notes: B = unstandardized coefficient; SE = standard error; OR = odds ratio; CI = confidence interval. standard deviations in parentheses for random effects.

**Table S16**

Logistic generalized linear mixed model (GLMM) with rule-breaking in part 2 as the dependent variable (comply=0, violate=1) and peer behavior (% of other breaking the rule), gender (male=0, female=1) and the interaction between peer behavior and age as independent variables, and with ‘participant’ as random intercept.

| Predictor | B | SE | OR | 95% CI for OR | p-value |
| --- | --- | --- | --- | --- | --- |
| Fixed effects |  |  |  |  |  |
| Intercept | -2.11 | 0.32 | 0.12 | [0.06, 0.23] | < .001 |
| Peer behavior | 0.05 | 0.00 | 1.05 | [1.05, 1.06] | < .001 |
| Gender | -2.80 | 0.46 | 0.06 | [0.02, 0.15] | < .001 |
| Peer behavior * Gender | 0.04 | 0.01 | 1.04 | [1.03, 1.05] | < .001 |
|  |  |  |  |  |  |
| Random effects |  |  |  |  |  |
| Variance of participant intercept (Level 2) | 13.47 (3.67) |  |  |  |  |

Notes: B = unstandardized coefficient; SE = standard error; OR = odds ratio; CI = confidence interval. standard deviations in parentheses for random effects.

**Table S17**

Logistic generalized linear mixed model (GLMM) with rule-breaking in part 1 as the dependent variable (comply=0, violate=1) and impulsivity as independent variable, and with ‘participant’ as random intercept.

| Predictor | B | SE | OR | 95% CI for OR | p-value |
| --- | --- | --- | --- | --- | --- |
| Fixed effects |  |  |  |  |  |
| Intercept | -2.20 | 0.52 | 0.11 | [0.04, 0.31] | < .001 |
| Impulsivity | 1.08 | 0.18 | 2.96 | [2.07, 4.22] | < .001 |
|  |  |  |  |  |  |
| Random effects |  |  |  |  |  |
| Variance of participant intercept (Level 2) | 2.02 (1.42) |  |  |  |  |

Notes: B = unstandardized coefficient; SE = standard error; OR = odds ratio; CI = confidence interval. standard deviations in parentheses for random effects.

**Table S18**

Logistic generalized linear mixed model (GLMM) with rule-breaking in part 1 as the dependent variable (comply=0, violate=1) and ambiguity (0=low, 1=high), impulsivity and the interaction between ambiguity and gender as independent variables, and with ‘participant’ as random intercept.

| Predictor | B | SE | OR | 95% CI for OR | p-value |
| --- | --- | --- | --- | --- | --- |
| Fixed effects |  |  |  |  |  |
| Intercept | -3.61 | 0.72 | 0.03 | [0.01, 0.11] | < .001 |
| Ambiguity | 2.39 | 0.83 | 10.93 | [2.13, 56.13] | .004 |
| Impulsivity | 1.43 | 0.25 | 4.19 | [2.57, 6.84] | < .001 |
| Ambiguity * Impulsivity | -0.47 | 0.28 | 0.62 | [0.36, 1.08] | .090 |
|  |  |  |  |  |  |
| Random effects |  |  |  |  |  |
| Variance of participant intercept (Level 2) | 2.86 (1.69) |  |  |  |  |

Notes: B = unstandardized coefficient; SE = standard error; OR = odds ratio; CI = confidence interval. standard deviations in parentheses for random effects.

**Table S19**

Logistic generalized linear mixed model (GLMM) with rule-breaking in part 1 as the dependent variable (comply=0, violate=1) and situational ambiguity (low=0, high=1) as independent variable, and with ‘class’ and ‘participant’ as random intercepts (h1a).

| Predictor | B | SE | OR | 95% CI for OR | p-value |
| --- | --- | --- | --- | --- | --- |
| Fixed effects |  |  |  |  |  |
| Intercept | 0.60 | 0.14 | 1.82 | [1.39, 2.39] | < .001 |
| Ambiguity | 1.00 | 0.16 | 2.72 | [1.98, 3.74] | < .001 |
|  |  |  |  |  |  |
| Random effects |  |  |  |  |  |
| Variance of class intercept (Level 2)  Variance of participant intercept (Level 3) | 0.02 (0.15)  2.98 (1.73) |  |  |  |  |

Notes: B = unstandardized coefficient; SE = standard error; OR = odds ratio; CI = confidence interval. standard deviations in parentheses for random effects.

**Table S20**

Logistic generalized linear mixed model (GLMM) with rule-breaking in part 1 as the dependent variable (comply=0, violate=1) and age (younger=0, older=1) as independent variable, and with ‘class’ and ‘participant’ as random intercepts (h1b).

| Predictor | B | SE | OR | 95% CI for OR | p-value |
| --- | --- | --- | --- | --- | --- |
| Fixed effects |  |  |  |  |  |
| Intercept | 0.98 | 0.16 | 2.66 | [1.95, 3.63] | < .001 |
| Age | 0.03 | 0.20 | 1.03 | [0.69, 1.52] | .894 |
|  |  |  |  |  |  |
| Random effects |  |  |  |  |  |
| Variance of participant intercept (Level 2)  Variance of class intercept (Level 3) | 2.19 (1.48)  0.03 (0.17) |  |  |  |  |

Notes: B = unstandardized coefficient; SE = standard error; OR = odds ratio; CI = confidence interval. standard deviations in parentheses for random effects.

**Table S21**

Logistic generalized linear mixed model (GLMM) with rule-breaking in part 1 as the dependent variable (comply=0, violate=1) and ambiguity (0=low, 1=high), age (younger=0, older=1) and the interaction between ambiguity and age as independent variables, and with ‘class’ and ‘participant’ as random intercepts (h1c).

| Predictor | B | SE | OR | 95% CI for OR | p-value |
| --- | --- | --- | --- | --- | --- |
| Fixed effects |  |  |  |  |  |
| Intercept | 0.51 | 0.19 | 1.66 | [1.14, 2.41] | .008 |
| Ambiguity | 1.17 | 0.23 | 3.24 | [2.06, 5.08] | < .001 |
| Age | 0.18 | 0.26 | 1.20 | [0.72, 1.99] | .478 |
| Ambiguity * Age | -0.33 | 0.30 | 0.72 | [0.40, 1.31] | .283 |
|  |  |  |  |  |  |
| Random effects |  |  |  |  |  |
| Variance of participant intercept (Level 2)  Variance of class intercept (Level 3) | 2.99 (1.73)  0.02 (0.15) |  |  |  |  |

Notes: B = unstandardized coefficient; SE = standard error; OR = odds ratio; CI = confidence interval. standard deviations in parentheses for random effects.

**Table S22**

Logistic generalized linear mixed model (GLMM) with rule-breaking in part 2 as the dependent variable (comply=0, violate=1) and peer behavior (% of others breaking the rule as independent variable, and with ‘class’ and ‘participant’ as random intercepts (h2a).

| Predictor | B | SE | OR | 95% CI for OR | p-value |
| --- | --- | --- | --- | --- | --- |
| Fixed effects |  |  |  |  |  |
| Intercept | -3.50 | 0.29 | 0.03 | [0.02. 0.05] | < .001 |
| Peer behavior | 0.07 | 0.00 | 1.07 | [1.06, 1.08] | < .001 |
|  |  |  |  |  |  |
| Random effects |  |  |  |  |  |
| Variance of participant intercept (Level 2)  Variance of class intercept (Level 3) | 11.88 (3.45)  0.74 (0.86) |  |  |  |  |

Notes: B = unstandardized coefficient; SE = standard error; OR = odds ratio; CI = confidence interval. standard deviations in parentheses for random effects.

**Table S23**

Logistic generalized linear mixed model (GLMM) with rule-breaking in part 2 as the dependent variable (comply=0, violate=1) and age (younger=0, older=1) as independent variable, and with ‘class’ and ‘participant’ as random intercepts (h2b).

| Predictor | B | SE | OR | 95% CI for OR | p-value |
| --- | --- | --- | --- | --- | --- |
| Fixed effects |  |  |  |  |  |
| Intercept | -0.48 | 0.15 | 0.62 | [0.46, 0.82] | .001 |
| Age | 0.89 | 0.20 | 2.43 | [1.63, 3.63] | < .001 |
|  |  |  |  |  |  |
| Random effects |  |  |  |  |  |
| Variance of participant intercept (Level 2)  Variance of class intercept (Level 3) | 3.65 (1.91)  0.06 (0.24) |  |  |  |  |

Notes: B = unstandardized coefficient; SE = standard error; OR = odds ratio; CI = confidence interval. standard deviations in parentheses for random effects.

**Table S24**

Logistic generalized linear mixed model (GLMM) with rule-breaking in part 2 as the dependent variable (comply=0, violate=1) and peer behavior (% of other breaking the rule), age (younger=0, older=1) and the interaction between peer behavior and age as independent variables, and with ‘class’ and ‘participant’ as random intercepts (h2c).

| Predictor | B | SE | OR | 95% CI for OR | p-value |
| --- | --- | --- | --- | --- | --- |
| Fixed effects |  |  |  |  |  |
| Intercept | -4.09 | 0.35 | 0.02 | [0.01, 0.03] | < .001 |
| Peer behavior | 0.07 | 0.00 | 1.07 | [1.06, 1.08] | < .001 |
| Age | 1.15 | 0.45 | 3.15 | [1.31, 7.59] | .010 |
| Peer behavior * Age | 0.01 | 0.01 | 1.01 | [1.00, 1.02] | .138 |
|  |  |  |  |  |  |
| Random effects |  |  |  |  |  |
| Variance of participant intercept (Level 2)  Variance of class intercept (Level 3) | 12.19 (3.49)  0.15 (0.39) |  |  |  |  |

Notes: B = unstandardized coefficient; SE = standard error; OR = odds ratio; CI = confidence interval. standard deviations in parentheses for random effects.

**2. Supplementary Figure: Distribution of responses in part 3**


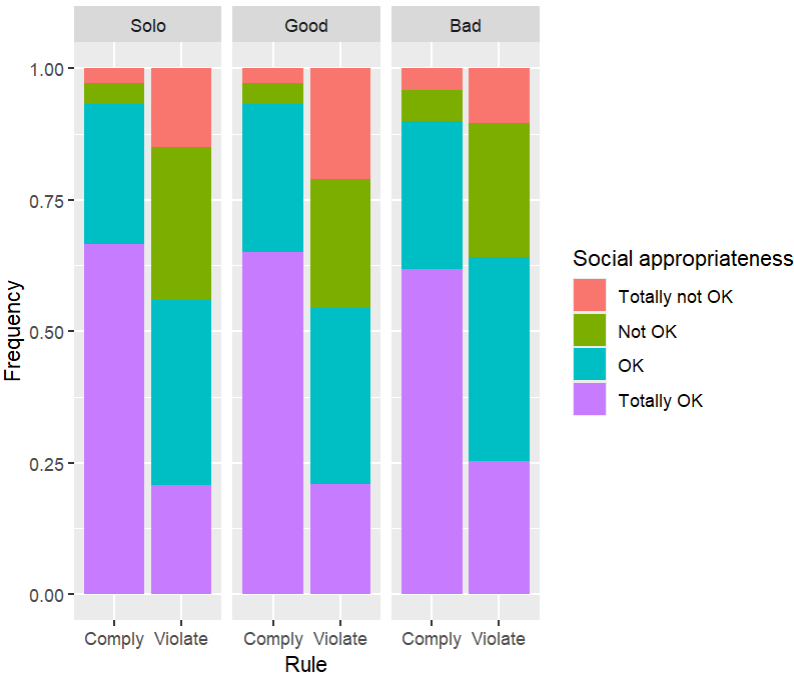


**Figure S1**: Distribution of social appropriateness rating of complying and violating in part 3 (for a summary of changes, see Fig. 4 of the main text). Solo contains responses before seeing the examples; Good shows the ratings after observing good examples (peers who follow the rule); Bad shows the ratings after observing bad examples (peers who break the rule).

**3. Supplementary Methods**

*Behavioral consistency across tasks*

In order to construct rule-breaking types, we first removed participants who, in part 1, broke the rule under low ambiguity, and followed the rule under high ambiguity (N = 49). We also removed participants who, in part 2, switched multiple times between rule-following and rule-breaking as the percentage of peers who broke the rule increased (N = 19, with N = 3 overlap with exclusions from part 1) and participants with inverted patterns (starting with rule-breaking when little peers break the rule, and changing to rule-following when more peer break the rule; N=17, no overlap with the exclusions from part 1). This leaves us with a sample of N = 551 participants.

For both parts, we then divided participants into three “types”: *consistent rule-followers* (who complied with the rule regardless of ambiguity or peer behavior; N = 115, and 123, respectively), *conditional rule-breakers* (who only broke the rule when ambiguity was high, N = 127, or who switched from complying to breaking the rule when a certain portion of others also breaks the rule, N = 300), and *consistent rule-breakers* (who always broke the rule regardless of ambiguity or peer behavior, N = 309, N = 128, respectively).

Besides the frequencies of the types for parts 1 and 2, as reported in the main text, we also explored if the types in part 1 and part 2 relate to the social appropriateness ratings before seeing examples in part 3, that is, do consistent rule-breakers think that compliance is less socially appropriate and violation is more socially appropriate than people that are conditional rule-breakers or consistent rule-followers. We found a significant difference in average social appropriateness rating of compliance by type in the behavioral task (part 1; F(1)=14.14, p < .001) and by type in the strategy method (part 2; F(1)=18.32, p < .001), that is, those who found compliance to be less socially appropriate are more likely to be a consistent rule-breaker. We also found a significant difference in average social appropriateness ratings of violation by type in the behavioral task (part 1; F(1)=168.5, p < .001) and by type in the strategy method (part 2; F(1)=115.90, p < .001), that is, those who found violation to be more socially appropriate are more likely to be consistent rule-breakers.

Furthermore, we explored if the conditional participants in part 2 (that is, people that switch between rule-following and rule-breaking at a certain percentage of others that break the rule and do not show inverted patterns, N = 300) are also the people that are more likely to change their injunctive norms after seeing examples. T-tests revealed that there was a significant effect of being socially sensitive on differences in social appropriateness rating of violation after seeing bad examples (M_conditional_ = 0.26, M_others_ = 0.04, t(281)=-2.74, p = .06). The effect was insignificant for differences in social appropriateness ratings of compliance after seeing good examples as well as bad examples The effect was also insignificant for differences in social appropriateness ratings of violation after seeing good examples.

**4. Exploratory Analyses: Impulsivity and resistance to peer influence**

At the end of the test battery, we included two questionnaires for exploratory purposes, namely the impulsive behavior short scale-8 (I-8; 𝛂 = . 75; Groskurth et al. 2022) and a questionnaire based on the Resistance to Peer Influence (RPI; 𝛂 = . 76) scale of Steinberg & Monahan (2007).

There was no significant difference in impulsivity score between young (M = 2.96, SD = 0.56; scale from 1 to 5) and old (M = 2.84, SD = 0.57) adolescents (t(624) = -0.52, p = .604). Impulsivity was significantly correlated positively with rule-breaking in the part 1 task with low ambiguity (r(628) = .24, p < .001) as well as rule-breaking in the version with high ambiguity (r(628) = .16, p < .001). There was no interaction effect between ambiguity and impulsiveness (B = -0.47, p = .090, OR = 0.62, 95% CI [0.36, 1.08]).

There was a significant difference in RPI score between young (M = 2.94, SD = 0.37; scale from 1 to 4) and old (M = 3.09, SD = 0.38) adolescents (t(624) = 5.07, p < .001). In the task with the strategy method (part 2) participants are exposed to different descriptive norms. Hence, RPI should matter in that specific task. However, we found no significant effect of RPI on rule-breaking in this task (B = -0.41, p = .088, OR = 0.67, 95% CI [0.42, 1.06]). We also found no significant interaction effect between RPI and descriptive norm (B = -0.00, p = .735, OR = 1.00, 95% CI [0.98, 1.01]). Furthermore, participants who had conditional strategies in part 2 had [lower] scores on the RPI scale (M = 3.00, SD = 0.35) than those who unconditionally broke or followed the rule (M = 3.06, SD = 0.42; t(546)=2.04, p=.042). These results indicate that sensitivity to peer influence in our task is related to self-reported RPI.

**5. Instruction protocol (translated from Dutch)**

| During data collection, the following protocol was used for oral instructions at the beginning of each experimental session. These sessions took place in the classroom of the participants. At the beginning of the session, tablets were handed out on which the participants completed the three experiments. |
| --- |

Hello and welcome everyone, nice that you are here. First of all, we will introduce ourselves. We are …………………………………. and we are researchers at the University of Amsterdam and today you are going to participate in our research about how you make decisions.

You are going to do some tasks and answer some questions on the tablets that are in front of you. In the tasks you can earn lottery tickets, the more tickets you earn, the bigger the chance you win the gift voucher that we are going to raffle off. This is a gift voucher of 20 euros. The more tickets you have, the more chances you thus have of winning the voucher.

It is important to know that the data that we collect is completely anonymous. We can therefore not see who does or fills in what. There are also no right or wrong answers in this experiment.

Before you start, you also give your own permission to participate in the research by means of a form. This is completely voluntary. If you do not want to participate, however, you cannot obtain the rewards. For those younger than 16, your parents have also given permission, Some parents have not yet given permission. We have a list here of those who do not have permission who can leave the room for a while and see if they can still contact their parents.

Each of you has a number on your desk. You must fill in this number for the 1^st^ question. This is your participant number. It is important that you fill in letters AND numbers. Also put your first name on the piece of paper. Because this is how we determine the winner of the gift voucher.

You will be able to read all the instructions about what exactly you have to do on the screen. If you have any questions, you can raise your hand and we will come and help you. When you are finished, raise your hand and we will come and collect the tablet. You can stay seated and do something for yourself for a while. Please stay quiet so that the other students can finish the experiment.

Are there any questions now?

Then you can start.

**6. Full battery of tasks and questionnaires (translated from Dutch)**

Welcome

Thank you for participating in our research.

Enter your participant number here. You will receive this number from us.

Start

—next screen—

In this research you will do **4 tasks**.

In these tasks you can win tickets for the lottery. The more tickets you win, the more chance you have of winning a gift voucher.

**The number of tickets you win depends on your choices**.

You will receive 5 tickets for participating.

Please note: do not close this screen or your browser. If you do, you will not be able to start over and you will not be able to win tickets!

Continue

—next screen—

Task 1

In this task you control a circle. You have to move this circle across the screen.

The circle starts on the left side of the screen. The circle moves to the traffic light and stops to wait.

You have to bring the circle across the finish line.

Example screen of the task:


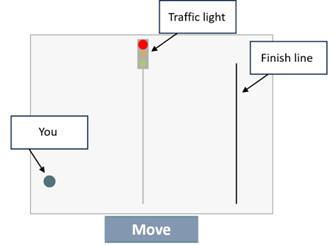


Continue

—next screen—

You decide when to cross with your circle.

You can wait to cross until the traffic light turns green.

Or you can cross before the traffic light turns green.

The rule is: **wait until the traffic light turns green**.

The number of tickets you earn with this task depends on your choice.

- If you **wait** until the traffic light turns green, you get **6 tickets**.

- If you **cross** before the traffic light turns green, you get **16 tickets**.

Back

Continue

—next screen—

We have done this task before, with 100 of your peers.

You will be presented with 5 situations.

For each of these situations, we ask you to make a choice:

**wait** until the light turns green, or **cross** before it turns green.

A. **10 out of 100 peers** **crossed** before the light turned green.

B. **30 out of 100 peers crossed** before the light turned green.

C. **50 out of 100 peers crossed** before the light turned green.

D. **70 out of 100 peers crossed** before the light turned green.

E. **90 out of 100 peers crossed** before the light turned green.

One of these situations actually happened.

Your choice for that situation determines how many tickets you earn for this task.

We will only tell you afterwards which one it was.

So think carefully about each of these choices!

Back

I understand the task. Continue

—next screen—

Before we start, let's check if you understand the task.

Indicate whether the following statements are **true** or **false**.

My choices determine how many tickets I earn.

True / False

For waiting for green, I get 6 tickets.

True / False

For crossing before it turns green, I get 16 tickets.

True / False

Back

Continue

—next screen—

Make your choices

The rule is: **wait until the traffic light turns green**.

**What would you do?**

For each situation, choose whether you are going to **wait** until the light turns green or **cross** before it turns green.

A. **10 out of 100 peers crossed** before the traffic light turned green.

Wait / Cross

B. **30 out of 100 peers crossed** before the traffic light turned green.

Wait / Cross

C. **50 out of 100 peers crossed** before the traffic light turned green.

Wait / Cross

D. **70 out of 100 peers crossed** before the traffic light turned green.

Wait / Cross

E. **90 out of 100 peers crossed** before the traffic light turned green.

Wait / Cross

Continue

—next screen—

You have completed the first task.

Well done!

Now we move on to the second task.

Continue

—next screen—

Task 2

In this task you will control the circle yourself.

You can also earn tickets in this task.

As you know: the more tickets, the greater the chance of a gift voucher.

You start this task with **20 tickets**

Continue

—next screen—

You control a circle that you can cross to the finish line.

If you press **move**, the circle moves to the traffic light. The circle will wait there.

If you press **move** again, the circle crosses to the finish line.

- Gif of the task –

Back

Continue

—next screen—

The rule is: **wait until the traffic light turns green**.

How many tickets you earn depends on how long you wait to cross.

You start with **20 tickets**. Every second in the task costs you **1 ticket**.

Once the circle has crossed the finish line, no more tickets are deducted.

Back

Continue

—next screen—

Task with pop-up screen:

Note:

Click Move to move your circle.

The rule is: wait until the traffic light is green.

OK

—next screen—

You've completed the second task. Great!

Now we're moving on to the third task.

Continue

—next screen—

Task 3

In this task you can earn 20 tickets.

How many tickets you earn depends on your answers, and the answers of your classmates.

Continue

—next screen—

This task is about what is "socially appropriate."

By this we mean what you think people generally see as "good," or "OK."

For example, if someone behaves in a socially inappropriate way, other people might get angry.

Back

Continue

—next screen—

Imagine someone is doing the traffic light task you just did.


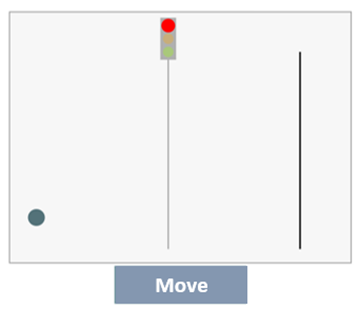


Just like you, they can choose to **wait** until the light turns green, or **cross** before it turns green.

Back

Continue

—next screen—

As before, the rule is: **wait until the traffic light turns green**.

The person can **wait** until it turns green or **cross** before it turns green.

Please indicate below what you think of these actions.

Please note - for each action: **if your answer is the same as that of a randomly selected classmate, you earn 5 extra tickets**.

The person **waits** until the traffic light turns green. He/she earns 6 tickets.

Totally not OK / Not OK / OK / Totally OK

The person **crosses** before the traffic light turns green. He/she earns 16 tickets.

Totally not OK / Not OK / OK / Totally OK

Continue

—next screen—

Now imagine: someone does the traffic light task that you just did.

But before this person makes his/her choice,

he/she first sees **what 6 other participants did**.

All 6 of these participants **crossed before the light turned green**.*

- Gif of the task with peers -

* Randomly assigned to one of two conditions. The other condition reads: All 6 of these participants waited until the light turned green

Continue

—next screen—

As before, the rule is: **wait until the traffic light turns green**.

The person can **wait** until it turns green or **cross** before it turns green.

Please indicate below what you think of these actions.

Please note - for each action: **if your answer is the same as that of a randomly selected classmate, you earn 5 extra tickets.**

The person waits until the traffic light turns green. He/she earns 6 tickets.

Totally not OK / Not OK / OK / Totally OK

The person crosses before the traffic light turns green. He/she earns 16 tickets.

Totally not OK / Not OK / OK / Totally OK

Back

Continue

—next screen—

You've completed the third task. Good job!

Now we move on to the fourth task.

Continue

—next screen—

Task 4

This task is very similar to Task 2 from earlier.

You will control the circle yourself again.

You can earn tickets again in this task.

As you know: the more tickets, the greater the chance of a gift voucher.

You start this task with **20 tickets**.

Continue

—next screen—

It works the same as in Task 2:

You control a circle that you can cross to the finish line.

If you press **move**, the circle moves to the traffic light. The circle will wait there.

If you press **move** again, the circle crosses to the finish line.

- Gif of the task with yellow light -

There is **one difference with Task 2**: the light turns **yellow** before it turns green

Back

Continue

—next screen—

The rule is: **wait until the traffic light turns green**.

How many tickets you earn depends on how long you wait to cross.

You start with **20 tickets**. Every second in the task costs you **1 ticket**.

Once the circle has crossed the finish line, no more tickets are deducted.

Back

Continue

—next screen—

Task with pop-up screen:

Note:

Click Move to move your circle.

The rule is: wait until the traffic light is green.

OK

—next screen—

You have completed the fourth task. Well done!

You are almost done!

We will end with some questions about yourself.

Continue

—next screen—

Questionnaire - screen 1 of 5

Please complete these questions

What is your gender?

Boy or male / Girl or female / Other or don’t want to say

What is your age?

10 / 11 / 12 / 13 / 14 / 15 / 16 / 17 / 18 / 19 / 20

What year of high school are you in?

First / Second / Third / Fourth / Fifth / Sixth

Continue

—next screen—

Questionnaire - screen 2 of 5

Below you will find four statements.

For each statement, indicate to what extent you think it is true for you.

Sometimes I do things impulsively that I shouldn't.

Completely not true / not true / neutral / true / completely true

To feel better, I sometimes do things that I later regret.

Completely not true / not true / neutral / true / completely true

I usually think carefully before I do something.

Completely not true / not true / neutral / true / completely true

I usually make decisions after careful and logical consideration.

Completely not true / not true / neutral / true / completely true

Continue

—next screen—

Questionnaire - screen 3 of 5

Yes! Your answers have been saved.

Below you will find four more statements.

For each statement, indicate to what extent you think it is true for you.

I always finish what I start.

Completely not true / not true / neutral / true / completely true

I manage my time well so that I can complete all my tasks on time.

Completely not true / not true / neutral / true / completely true

I am prepared to take risks.

Completely not true / not true / neutral / true / completely true

I like to take risks.

Completely not true / not true / neutral / true / completely true

Continue

—next screen—

Questionnaire - screen 4 of 5

Thank you. Your answers have been saved.

Below you will see five descriptions of how you can respond to certain situations.

Indicate to what extent the description fits you.

I do what my friends want to do, just to keep my friends happy.

Completely disagree / disagree / agree / completely agree

I find it more important to be an individual, than to belong to the group.

Completely disagree / disagree / agree / completely agree

I am easily persuaded by my friends.

Completely disagree / disagree / agree / completely agree

I do things that I know are wrong, just to keep my friends happy.

Completely disagree / disagree / agree / completely agree

I hide my real opinions from my friends because I think my friends would laugh at me for it.

Completely disagree / disagree / agree / completely agree

Continue

—next screen—

Questionnaire - screen 5 of 5

You're almost there!

Below you will see five more descriptions of how you can react to certain situations.

Indicate to what extent the description fits you.

I would break the law because my friends say they would.

Completely disagree / disagree / agree / completely agree

I change myself so much when I'm with my friends that I wonder who I really am.

Completely disagree / disagree / agree / completely agree

I take more risks when I'm with my friends than when I'm alone.

Completely disagree / disagree / agree / completely agree

I say things I don't really believe because I think my friends will respect me more.

Completely disagree / disagree / agree / completely agree

I think it's better to be an individual, even if people will be angry with me for going against the group.

Completely disagree / disagree / agree / completely agree

Continue

—next screen—

This is the end of the research!

These are your earnings:

For participating, you will receive 5 tickets.

Task 1: the real number of the 100 previous participants who crossed before the green was 30.

For that situation, you choose …

Earnings: X tickets.

Task 2: when you crossed the finish line, you had X tickets left.

Task 3: we compared your 4 answers to the question "how OK was it to (not) wait for the green"

with the answers of your classmates. Of the 4 answers, X were the same.

Earnings: X tickets.

Task 4: when you crossed the finish line, you had 16X tickets left.

Your total number of tickets is X.

Raise your hand, and sit quietly for a moment.

Your tablet will be picked up shortly.

One of us will draw the winning ticket and announce the winner.

Thank you for your participation!
